# Multiferroicity in atomic van der Waals heterostructures

**DOI:** 10.1038/s41467-019-10693-0

**Published:** 2019-06-14

**Authors:** Cheng Gong, Eun Mi Kim, Yuan Wang, Geunsik Lee, Xiang Zhang

**Affiliations:** 1 Nano-scale Science and Engineering Center (NSEC), 3112 Etcheverry Hall, University of California, Berkeley, CA 94720 USA; 2 Department of Chemistry, Ulsan National Institute of Science and Technology, Ulsan, 44919 Korea; 3 Materials Sciences Division, Lawrence Berkeley National Laboratory, 1 Cyclotron Road, Berkeley, CA 94720 USA

**Keywords:** Information storage, Magnetic properties and materials, Two-dimensional materials

## Abstract

Materials that are simultaneously ferromagnetic and ferroelectric – multiferroics – promise the control of disparate ferroic orders, leading to technological advances in microwave magnetoelectric applications and next generation of spintronics. Single-phase multiferroics are challenged by the opposite *d*-orbital occupations imposed by the two ferroics, and heterogeneous nanocomposite multiferroics demand ingredients’ structural compatibility with the resultant multiferroicity exclusively at inter-materials boundaries. Here we propose the two-dimensional heterostructure multiferroics by stacking up atomic layers of ferromagnetic Cr_2_Ge_2_Te_6_ and ferroelectric In_2_Se_3_, thereby leading to all-atomic multiferroicity. Through first-principles density functional theory calculations, we find as In_2_Se_3_ reverses its polarization, the magnetism of Cr_2_Ge_2_Te_6_ is switched, and correspondingly In_2_Se_3_ becomes a switchable magnetic semiconductor due to proximity effect. This unprecedented multiferroic duality (i.e., switchable ferromagnet and switchable magnetic semiconductor) enables both layers for logic applications. Van der Waals heterostructure multiferroics open the door for exploring the low-dimensional magnetoelectric physics and spintronic applications based on artificial superlattices.

## Abstract (web summary)

Low dimensional multiferroic materials promise the technological advances in next generation spintronic and microwave magnetoelectric devices. Here the authors propose the multiferroicity in the atomically thin ferromagnetic Cr2Ge2Te6/ferroelectric In2Se3 van der Waals heterostructure due to the crosslayer magnetoelectric coupling.

## Introduction

Multiferroics, a class of functional materials that simultaneously possess more than one ferroic orders such as ferromagnetism and ferroelectricity, hold great promise in magnetoelectric applications due to the inherent coupling between ferroic orders^1–6^, leading to technological advances in next generation of spintronics and microwave magnetoelectric devices. However, single-phase multiferroics are challenged by the different ferroics’ contradictory preference on the d-orbital occupation of metal ions: ferroelectricity arising from off-center cations requires empty *d*-orbitals, whereas ferromagnetism usually results from partially filled *d*-orbitals^7^. Conventional perovskite multiferroics (chemical formula: ABO_3_) have lone-pair-active A-sites which move to off-centers of centrosymmetric crystals for electric polarization, and B-sites with unpaired electrons for magnetic order. Because the ferroelectric and magnetic order in these materials are associated with different ions, the coupling between the ferroic orders are usually weak.

Heterogeneous multiferroics, synthesized composites of two mixed phases^8^, have the coupling between ferroelectric and magnetic order exclusively at inter-materials boundaries, with magnetoelectric effects occasionally established via interfacial magnetoelastic effect. As an example, magnetic nanopillars could be embedded in ferroelectric matrix. However, these heterogeneous multiferroics stringently demand the constituent materials on their structural similarity, lattice match and chemical compatibility, and have weak magnetoelectric effects limited by the interface/bulk ratios.

Van der Waals (vdW) crystals emerged as ideal material systems with unprecedented freedom for heterostructure construction^9^. Recent experimental advance discovered ferromagnetism^10–12^ and ferroelectricity^13^ in different two-dimensional vdW crystals separately. It remains a paramount challenge to realize multiple ferroic orders in a single-phase 2D material simultaneously^14–17^, as each order encounters its own challenge (e.g., ferromagnetism in 2D systems suffers from enhanced thermal fluctuations, whereas ferroelectricity the depolarization field). Constructing heterostructures of 2D magnets and 2D ferroelectrics potentially provides a generally applicable route to create 2D multiferroics. However, the fundamental question remains regarding whether the interlayer magnetoelectric coupling can be established, given the presence of the interlayer vdW spacing. If realized, layered heterostructure multiferroics would provide completely new platforms with all atoms participating in the inter-ferroics coupling, and largely reshape the landscape of multiferroics based on vdW superlattices.

Through first-principles density functional theory (DFT) calculations based on a bilayer heterostructure of ferromagnetic Cr_2_Ge_2_Te_6_ and ferroelectric In_2_Se_3_ monolayers^18–21^, we discovered a strong interlayer magnetoelectric effect: the reversal electric polarizations in In_2_Se_3_ switches the magnetocrystalline anisotropy of Cr_2_Ge_2_Te_6_ between out-of-plane and in-plane orientations. For a 2D ferromagnet, such a change in magnetic anisotropy corresponds to a switching on/off of the ferromagnetic order at finite temperatures, for easy-axis anisotropy opens spin wave excitation gap and thus suppresses the thermal fluctuations, but easy-plane anisotropy does not^10,22,23^. The switching of ferromagnetic order by electric polarization promises a novel design of magnetic memory. Detailed analysis unraveled the interfacial hybridization as the cause of interlayer magnetoelectric coupling. Furthermore, In_2_Se_3_ becomes magnetized due to the proximity to Cr_2_Ge_2_Te_6_, making In_2_Se_3_ a single-phase multiferroics (i.e., ferromagnetic and ferroelectric orders coexist in In_2_Se_3_), although apparently the magnetization of In_2_Se_3_ requires the presence of the adjacent Cr_2_Ge_2_Te_6_. Such multiferroicity duality—that is, the interlayer multiferroicity and the In_2_Se_3_ intralayer multiferroicity—provides unique solid-state system in which ferroelectric and ferromagnetic orders interplay inherently. This unusual multiferroicity duality in vdW heterostructures may open avenues for developing new concepts of magnetoelectric devices: using single knob (the orientation of electric polarization in In_2_Se_3_) to control the magnetic order in both In_2_Se_3_ and Cr_2_Ge_2_Te_6_. We envision the multiferroicity duality potentially enriches the freedom of layer-resolved data storage and that of information processing due to the diverse magnetoelectric and magneto-optic properties of constituent layers.

## Results

### Material model and computational details

**Fig. 1:**
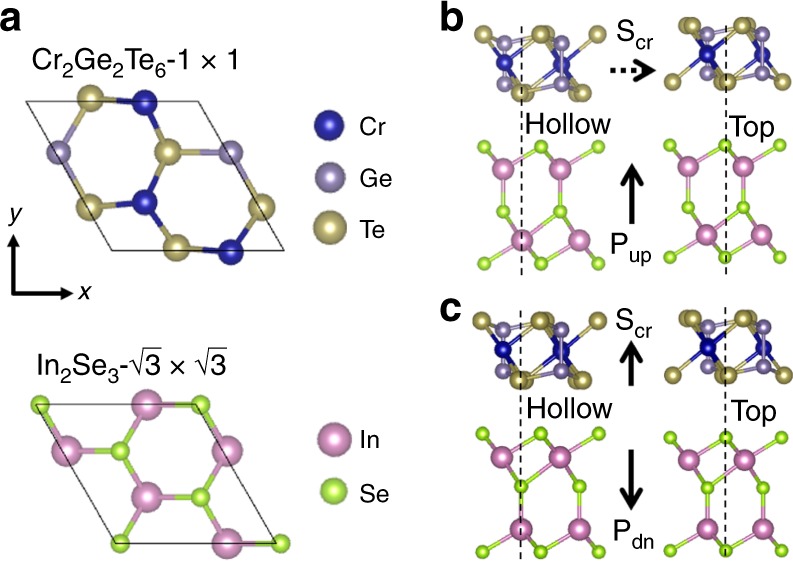
Van der Waals Cr_2_Ge_2_Te_6_/In_2_Se_3_ heterostructure and magnetoelectric coupling. **a** Lateral unit cell size is chosen to be equal to that of ferromagnetic Cr_2_Ge_2_Te_6_ experimental lattice constant and commensurate to ferroelectric In_2_Se_3_-$\sqrt 3 \, \times \, \sqrt 3$ with −4% strain. The interfacial two atomic layers (one atomic layer from each side of the interface) are shown in the top views of Cr_2_Ge_2_Te_6_-1 × 1 (top) and In_2_Se_3_-$\sqrt 3 \, \times \, \sqrt 3$ (bottom). **b**, **c** Heterostructure side views with the In_2_Se_3_ ferroelectric dipole moment directed upward and downward (**P**_**up**_ and **P**_**dn**_), respectively, and the induced easy-plane and easy-axis Cr spins (**S**_**Cr**_). The solid (dashed) arrow of **S**_**Cr**_ indicates the allowed (prohibited) finite temperature long-range ferromagnetic ordering in 2D systems by the Mermin-Wagner theorem. For each case, the most stable (hollow) and unstable (top) stacking configurations are given

In this work, the lattice constant of Cr_2_Ge_2_Te_6_ adopted the experimental value 6.83 Å and was fixed in heterostructures for the sake of minimizing artifact effects, considering the magnetic properties of 2D Cr_2_Ge_2_Te_6_ are sensitive to structure parameters. It has been reported that a monolayer In_2_Se_3_ of either zincblende or wurtzite stacking is unstable with a tendency of the lateral displacement of the top Se layer, leading to the energetically degenerate ferroelectric monolayer^18^. Although the one relaxed from the zincblende stacking is chosen in this study, it will be also applicable to the other derived from the wurtzite, because the main mechanism to be shown is determined by the interfacial monolayers and thus independent of the detailed stacking type of the multilayer In_2_Se_3_. The optimized lattice constant of 1 × 1-In_2_Se_3_ (4.106 Å), is strained by −4.0% to match In_2_Se_3_-$\sqrt 3 \, \times \,\sqrt 3$ to Cr_2_Ge_2_Te_6_-1 × 1 as shown by Fig. 1a. In heterostructure, the relative spacing and registry between Cr_2_Ge_2_Te_6_ and In_2_Se_3_ are adjusted to find the energy minimum configuration. The reversal of the electric polarization of the isolated In_2_Se_3_ monolayer can be achieved via lateral displacement of the middle most Se layer, with an energy barrier as small as 0.04 eV per formula unit estimated by nudged elastic band calculation^24^. In heterostructure, due to the large vdW spacing, the presence of Cr_2_Ge_2_Te_6_ does not noticeably affect the energy barrier of the electric-polarization reversal process of In_2_Se_3_. The total energy of the heterostructure is lowest (highest) where the interfacial Te atoms sit at the hollow (top) site of In_2_Se_3_, with their relative energy difference amounts to 0.31 and 0.35 eV/u.c. for upward (Fig. 1b) and downward (Fig. 1c) polarizations, respectively. The equilibrium interlayer distance between Cr_2_Ge_2_Te_6_ and In_2_Se_3_ at the hollow configuration is 3.20 and 3.14 Å for up and down polarizations of In_2_Se_3_, respectively. The total energy of the down polarization (Fig. 1c) is lower by 0.07 eV/u.c. than that of the up polarization (Fig. 1b), due to the stronger interfacial coupling between downpolarized In_2_Se_3_ and Cr_2_Ge_2_Te_6_.

In order to reproduce the experimental magnetic properties of bulk Cr_2_Ge_2_Te_6_, we used small onsite Hubbard *U* value 0.5 eV and Hund’s coupling *J* value 0.0 eV for Cr *d* orbital in DFT calculations (see ref. ^10^ for the choice of *U* = 0.5 eV, *J* = 0.0 eV). This small onsite Columbic interaction is consistent with the fact that Cr_2_Ge_2_Te_6_ is a small band gap material with less localization than Cr-oxides. The ferromagnetic ground state is confirmed with the Cr spin magnetic moment ~3.0 μ_B_. With the spin–orbit coupling (SOC) included, the magnetocrystalline anisotropy energy (MAE) is calculated and defined as $E_{[100]} - E_{[001]}$, where the former and latter correspond to the total energy with the Cr spins directed in-plane and out-of-plane, respectively. Due to the threefold rational symmetry of Cr_2_Te_2_Te_6_, there is not much magnetic anisotropy within the basal plane. We checked the convergence of MAE carefully, where a large value of K-mesh (12 × 12 × 1) was enough to ensure the error <10 μeV/Cr. For the isolated monolayer Cr_2_Ge_2_Te_6_, our calculated MAE is −70 μeV/Cr, favoring the in-plane direction. In the heterostructures with up- and downpolarized In_2_Se_3_, the calculated Cr MAE is −95 and 75 μeV, respectively, whose energetically favorable spin orientations are indicated by **S**_Cr_ in Fig. 1b, c. By modulating the polarization of the adjacent ferroelectric layer, the switching of the magnetization orientation is realized. This has significant application implications: For a 2D magnetic system with easy-plane anisotropy (X–Y model), the finite temperature ferromagnetic order is inhibited, whereas for easy-axis anisotropy (Ising model), the magnetic order can be sustained at finite temperatures. Therefore, in such a heterostructure, multiferroic effect offers a potential route to switch the ferromagnetism for logic devices.

### Mechanism for interfacial multiferroicity

The mechanism for electric-polarization dependent MAE is discussed in details. The calculated Cr orbital moment is small (<L_x_> = 0.04 μ_B_, <L_z_> = 0.01 μ_B_ for in-plane, out-of-plane spin directions), which is less likely the origin of MAE. The plausible mechanism is related to the detailed feature of spin-resolved orbital-decomposed band structure^25^. Starting from the collinear spin band structures, we analyzed the energy correction by the perturbation theory about λ**L**∙**S** where λ is the radial part of Cr SOC. The orbital moment quenched by the crystal field results in the vanishing first order correction. Assuming the negligible change of the electron correlation energy between [100] and [001] spin directions, one can write the second order contribution to MAE as follows^25^:

1 $${\mathrm{MAE}} =	 \lambda ^2\mathop {\sum }\limits_{v,c,\sigma } \left( {\left| \langle{v,\sigma {\mathrm{|}}L_z{\mathrm{|}}c,\sigma }\rangle \right|^2 - \left| \langle{v,\sigma {\mathrm{|}}L_x{\mathrm{|}}c,\sigma }\rangle \right|^2} \right)/\left( {{\it{\epsilon }}_{c,\sigma } - {\it{\epsilon }}_{v,\sigma }} \right) \\ 	 + \, \lambda ^2\mathop {\sum }\limits_{v,c,\sigma \ne \sigma \prime } \left( {\left|\langle {v,\sigma {\mathrm{|}}L_x{\mathrm{|}}c,\sigma \prime } \rangle\right|^2 - \left|\langle {v,\sigma {\mathrm{|}}L_z{\mathrm{|}}c,\sigma \prime }\rangle \right|^2} \right)/\left( {{\it{\epsilon }}_{c,\sigma \prime } - {\it{\epsilon }}_{v,\sigma }} \right),$$

Here the first and second summations correspond to the spin-conserving |Δ*s*_z_| = 0 and spin-flipping |Δ*s*_z_| = 1 transitions, and $|v,\sigma\rangle$ and $|c,\sigma\rangle$ are valence and conduction band states with spin *σ*, respectively, whose energy eigenvalues are ${\it{\epsilon }}_{c,\sigma }$ and ${\it{\epsilon }}_{v,\sigma }$. The angular momentum matrix elements of Lz and Lx correspond to transitions with |Δ*m*_z_| = 0 and |Δ*m*_z_| = 1, respectively, for Cr *d*-orbitals. Therefore, for spin-conserving transition, SOC elements between occupied and unoccupied states with the same (different) magnetic quantum number through the $L_z\left( {L_x} \right)$ operator contributes to positive (negative) MAE. For spin-flipping transition, the contribution to MAE is reversed^26^.

**Fig. 2:**
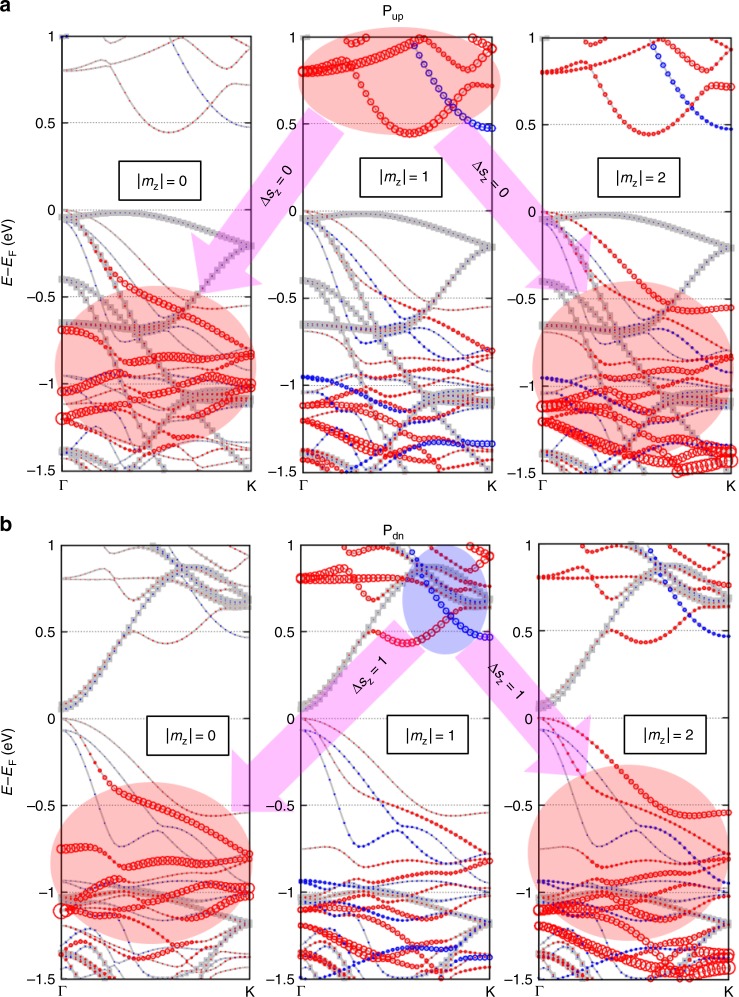
Spin-polarized and Cr *d* orbital-decomposed band structures of Cr_2_Ge_2_Te_6_/In_2_Se_3_ heterostructures. **a**, **b** The calculated band structures for the heterostructures with **P**_**up**_ and **P**_**dn**_ In_2_Se_3_, respectively. For each electric polarization, the heterostructure (in terms of contact registry and interlayer distance) adopted the configuration of global energy minimum (see Supplementary Fig. 1). The majority and minority spin states are indicated by the red and blue circles, respectively, whose size denotes the contribution of Cr *d* orbitals with certain azimuthal angular momentum |*m*_z_|. In_2_Se_3_ states are shown by the gray dots, and the band exhibits the overall shift down by ~1 eV while changing In_2_Se_3_ from **P**_**up**_ and **P**_**dn**_, which indicates a strong In_2_Se_3_-polarization dependent interfacial coupling. The pink arrows indicate the SOC elements between empty and filled states, indicated by the red circle for the majority spin or blue for minority, causing the negative or positive value of MAE (see Eq. (1) and text) for (**a**) or (**b**), respectively, thus the easy-plane or easy-axis **S**_**Cr**_

Figure 2a, b shows the spin-resolved orbital-decomposed band structure of heterostructures for up- and downpolarized In_2_Se_3_, respectively, where the contribution of Cr *d*-orbitals is indicated by the circles for |*m*_z_| = 0 (*z*^2^), 1 (*xz* and *yz*), or 2 (*x*^2^−*y*^2^ and *xy*). As shown by the arrows in Fig. 2a for the heterostructure with up-polarized In_2_Se_3_, our calculated negative MAE mainly originates from the spin-conserving transition from |*m*_z_| = 1 to |*m*_z_| = 0 or 2, i.e., |Δ*s*_z_| = 0 and |Δ*m*_z_| = 1 related to the second term of the first sum in Eq. (1). The mechanism is further confirmed by our results that the Cr MAE changes from about −100 to 200 μeV by intentionally increasing the *U* value from 0.5 to 2.0 eV: the increased *U* lowers the energy level of the majority spin in valence bands of |*m*_z_| = 0 or 2 (Supplementary Fig. 3), and thus the transition energy gap of |Δ*s*_z_| = 0 and |Δ*m*_z_| = 1 is increased and the associated contribution is weakened, leading to the positive MAE.

However, for the heterostructure with downpolarized In_2_Se_3_, as shown in Fig. 2b, the conduction band minimum of Cr *d* |*m*_z_| = 1 shows a large gap (~0.2 eV) near 0.5 eV above Fermi level, which is caused by hybridization with the In_2_Se_3_ conduction band minimum. Such hybridization results in a significant depletion of |*m*_z_| = 1 majority spin DOS (Supplementary Fig. 2a). Hence, the negative contribution to MAE found for the case of up-polarized In_2_Se_3_ is suppressed. Meanwhile the minority spin DOS remains almost unchanged for |*m*_z_| = 1 (Supplementary Fig. 2a), leading to positive MAE via |Δ*s*_z_| = 1 and |Δ*m*_z_| = 1, as illustrated by the arrows in Fig. 2b. Considering the interfacial hybridization depends on the band alignment of In_2_Se_3_ and Cr_2_Ge_2_Te_6_, we employed the Heyd-Scuseria-Ernzerhof exchange-correlation functional (HSE06) to recalculate the band properties of the heterostructures. As expected, the calculated band gaps widen compared with the GGA-PBE results, but the key features while In_2_Se_3_ reverses its electric orientation from up to down keep the same: the conduction band of In_2_Se_3_ moves down to hybridize with the conduction band of Cr_2_Ge_2_Te_6_, as clearly seen in Supplementary Fig. 4.

**Fig. 3:**
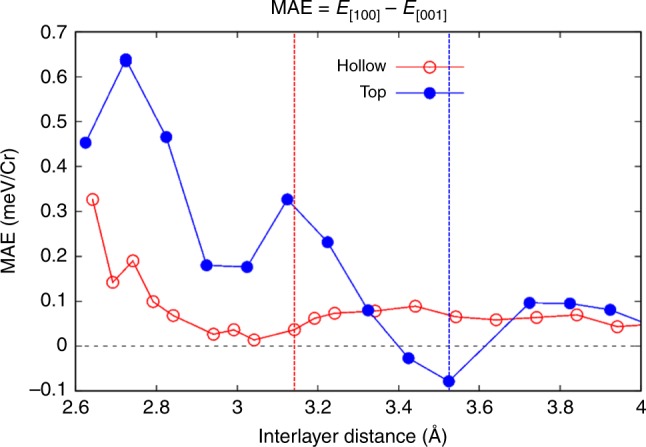
Calculated magnetocrystalline anisotropy of Cr_2_Ge_2_Te_6_ in the heterostructure versus the vdW interlayer distance. Magnetocrystalline anisotropy energy (MAE) (in meV/Cr) of Cr_2_Ge_2_Te_6_ as a function of the distance to In_2_Se_3_ in the **P**_**dn**_ state, showing a gradual increase towards the positive MAE (easy-axis **S**_**Cr**_) upon decreasing the distance for both hollow and top stacking configurations. This set of calculations confirmed the scenario that the enhanced interfacial hybridization between Cr_2_Ge_2_Te_6_ and In_2_Se_3_ tends to switch the magnetocrystalline anisotropy from easy-plane to easy-axis. The calculation error is reflected by the symbol size (~0.01 meV/Cr). The red and blue dashed lines correspond to the equilibrium interfacial distances for hollow and top stacking configurations, respectively

The sign change of Cr_2_Ge_2_Te_6_’s MAE upon the electric-polarization reversal of In_2_Se_3_ from up to down arises from the increased coupling, which causes the overall shift down of In_2_Se_3_ bands and its enhanced hybridization with Cr_2_Ge_2_Te_6_. This suggests that the positive MAE for the down polarization would be enhanced by a reduced vdW spacing. To confirm this scenario, we did interlayer spacing dependent MAE calculations for hollow configuration as shown in Fig. 3. As the interlayer distance decreases, MAE increases gradually with a slight fluctuation. The fluctuation originates from the detailed variation of energy levels in the spin-polarized band structures. From the same calculation conducted for the top configuration, it exhibits a stronger fluctuation with the interlayer spacing, due to the larger degree of interfacial orbital overlap in top configuration. The same trend of the two curves in Fig. 3 confirms that the increased interlayer hybridization tends to switch the magnetocrystalline anisotropy from easy-plane to easy-axis. Detailed spin-resolved orbital-decomposed analysis in Supplementary Fig. 5 shows that the decreased spin-flipping energy gap near K with decreased interlayer distance contributes to the positive MAE.

### Magnetized In_2_Se_3_ in proximity to Cr_2_Ge_2_Te_6_

**Fig. 4:**
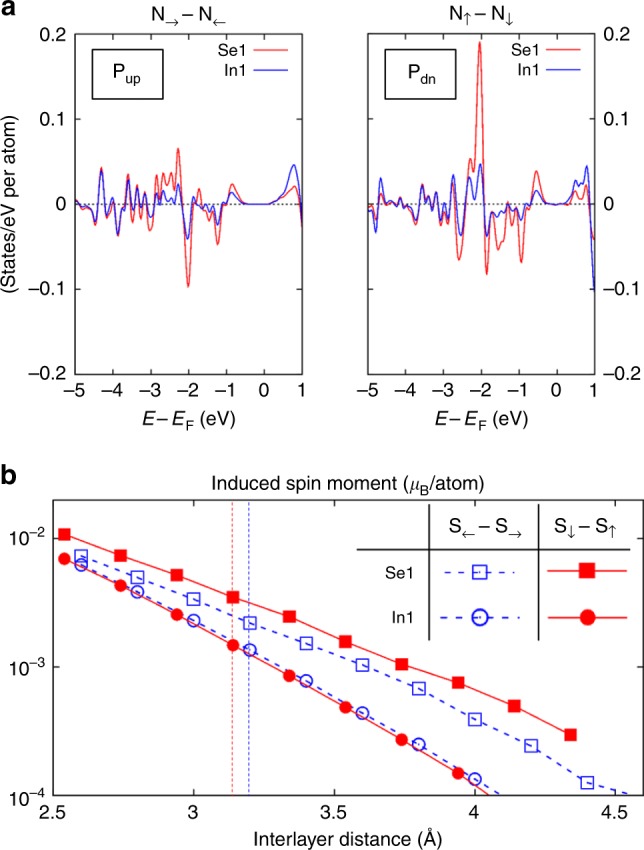
Magnetoelectric effect in In_2_Se_3_, mediated by the magnetic proximity to Cr_2_Ge_2_Te_6_. **a** Projected spin density of states for the surface (Se1) and subsurface (In1) atomic layers close to Cr_2_Ge_2_Te_6_. Left and right panels correspond to the heterostructures with **P**_**up**_ and **P**_**dn**_ In_2_Se_3_, respectively, with the in-plane and out-of-plane spin quantization axis. Given the prohibited long-range magnetic order for the easy-plane 2D magnetic system, the spin-polarized In_2_Se_3_ as shown by the left panel of (**a**) is only a ground state at zero Kelvin. But the easy-axis spin-polarized In_2_Se_3_ as shown by the right panel of (**a**) is practical at finite temperatures. Hence, the magnetization of the semiconducting In_2_Se_3_ can be switched by its own electric polarization, while in proximity to Cr_2_Ge_2_Te_6_. **b** Interlayer distance dependence of the proximity-induced Se1 and In1 spin moments. The vertical dashed lines denote the equilibrium distances predicted by PBE with D2 vdW correction. Note that the induced spin moments are negative, which means the interlayer antiferromagnetic proximity effect in the studied In_2_Se_3_-Cr_2_Ge_2_Te_6_ heterostructures

Remarkably, the exchange splitting in Cr *d*-band magnetizes In_2_Se_3_ by the proximity effect. As shown in Fig. 2, the highest valence band has a significant exchange splitting, where the majority-spin band is closer to the Fermi level near Γ. Those states are mainly caused by the surface Te atoms in Cr_2_Ge_2_Te_6_, which means the interfacial Te atoms have the electron spin antiparallel to that of Cr *d* electrons. Our calculated spin moment per Te atom is −0.11 μ_B_for In_2_Se_3_ of either electric polarizations. Also, the surface In and Se atoms has non-zero spin moments parallel to Te spins induced by the proximity. The spin-resolved DOS of interfacial In and Se, shown in Fig. 4a, confirmed the magnetized In_2_Se_3_. It is practically important to note the calculated magnetized In_2_Se_3_ here is a ground-state property. At finite temperatures, easy-plane magnetization of 2D In_2_Se_3_ is susceptible to thermal fluctuations and long-range order does not exist, but easy-axis magnetization of 2D In_2_Se_3_ could sustain the spin polarization at certain finite temperatures. Hence, a switching of 2D magnetic ferroelectric In_2_Se_3_ could be realized in this heterostructure multiferroics, leading to a design of spin field-effect transistor^27,28^.

The induced spin moment of surface Se is attributed to the exchange coupling *J* ~ *t*^2^/*U* between Te *p* and Se *p* orbitals, with *t* the hopping constant and *U* the intra-orbital Coulomb repulsion. In the limit of zero *t* or infinite *U*, the system favors the triplet state similar to the atomic Hund coupling, which is the case for Te *p* and Se *p*. For a given value of *U*, the *t* varies exponentially with the distance. Consistently, our calculations show the exponentially increasing Se spin moment with decreased interfacial distance, as shown in Fig. 4b. The correlation effect should depend on the specific nonlocal correlation functional. For different vdW functionals, the induced spin moments remain at nearly the same magnitude (Supplementary Fig. 6).

### Discussion on practical experimental factors

It is of experimental guidance to remark on the possible effects of real material environments. Calculation and analysis in this work are based on the heterostructure of a bilayer system floating in vacuum. In the experimental realization, the initial anisotropy of the magnetic layer Cr_2_Ge_2_Te_6_ could be affected by a few factors, including contacting the materials of large dielectric constants^22,29^ or large SOC strengths^30^, unintentional doping^31–33^ caused by chemicals in device fabrication process, and small amount of artificial strain induced in heterostructure preparation. These factors may affect the resultant magnetoelectric effect quantitatively, as reflected from the calculated MAE of the heterostructures based on the arbitrary sets of *U* and *J* values (see Supplementary Fig. 7): the increased *U* value enhances the out-of-plane anisotropy, while the increased *J* value enhances the in-plane anisotropy; for any tested set of *U* and *J* values, the out-of-plane anisotropy is always enhanced by ~ 0.15 meV/Cr when the In_2_Se_3_ dipole is inverted from up to down. Therefore, even if our adopted values of *U* and *J* (*U* = 0.5 eV, *J* = 0.0 eV) slightly deviate from the exact description of the real heterostructure samples because of the aforementioned complex experimental conditions, the reversal of the In_2_Se_3_ polarization from up to down always strengthens the 2D ferromagnetic order in Cr_2_Ge_2_Te_6_. This leads to a general implication: in practice, one can always set a temperature so that 2D ferromagnetism could be found in P_dn_-In_2_Se_3_-Cr_2_Ge_2_Te_6_ but disappear from P_up_-In_2_Se_3_-Cr_2_Ge_2_Te_6_, leading to the practical switching experiments at finite temperatures. Therefore, the magnetoelectric effect presented here, based on the modification of MAE by the intricate interface hybridization which further relates to the electric polarization of the 2D ferroelectrics, is an intrinsic interfacial phenomenon.

### Summary

We employed first-principles DFT calculations on a vdW heterostructure consisting of ferromagnetic Cr_2_Ge_2_Te_6_ and ferroelectric In_2_Se_3_ monolayers. By reversing the electric polarization of In_2_Se_3_, the calculated magnetocrystalline anisotropy of Cr_2_Ge_2_Te_6_ changes between easy-axis and easy-plane (i.e., switching on/off of the ferromagnetic order), which promises a novel design of magnetic memory. Furthermore, In_2_Se_3_ becomes magnetic ferroelectrics, with switchable spin polarizations according to its own electric polarization. The 2D multiferroic heterostructures would tremendously enlarge the landscape of multiferroics by artificially assembling 2D layers and provide new material platforms for a plethora of emergent interfacial phenomena.

## Methods

### The DFT method and parameters

All the calculations were performed by the DFT method implemented in Vienna ab initio Simulation Package (VASP)^34^, with the Perdew-Burke-Ernzerhof (PBE) functional^35^ in the scheme of generalized gradient approximation (GGA). The main data was calculated by GGA + U based on the Liechtenstein approach with *U* = 0.5 eV and *J* = 0.0 eV. The van der Waals interatomic forces are described by the D2 Grimme method^36^. The K-mesh of 6 × 6 × 1 and the energy cutoff of 300 eV are used for the structural optimization. The dipole correction is included to exclude spurious dipole–dipole interaction between periodic images.

## Supplementary information

Supplementary Info

## Data Availability

The data that support the findings of this study are available from the corresponding author upon reasonable request.
