# Supplementary material for: Multiferroicity in atomic van der Waals heterostructures
Source: Nat Commun. 2019 Jun 14;10:2657. doi: 10.1038/s41467-019-10693-0 (PMC6570651; doi:10.1038/s41467-019-10693-0)
Supplement: Supplementary file 1 — Supplementary Info [file 41467_2019_10693_MOESM1_ESM.pdf]

Supplementary Information

Multiferroicity in atomic van der Waals heterostructures

Cheng Gong,[1] Eun Mi Kim,[2] Yuan Wang,[1,3] Geunsik Lee,[2] Xiang Zhang[1,3]

[1]Nano-scale Science and Engineering Center (NSEC), 3112 Etcheverry Hall, University of California, Berkeley, California 94720, USA

[2]Department of Chemistry, Ulsan National Institute of Science and Technology, Ulsan 44919, Korea

[3]Materials Sciences Division, Lawrence Berkeley National Laboratory, 1 Cyclotron Road, Berkeley, California 94720, USA

Correspondence and requests for materials should be addressed to G.L. (email: gslee@unist.ac.kr) or to X.Z. (email: xiang@berkeley.edu)

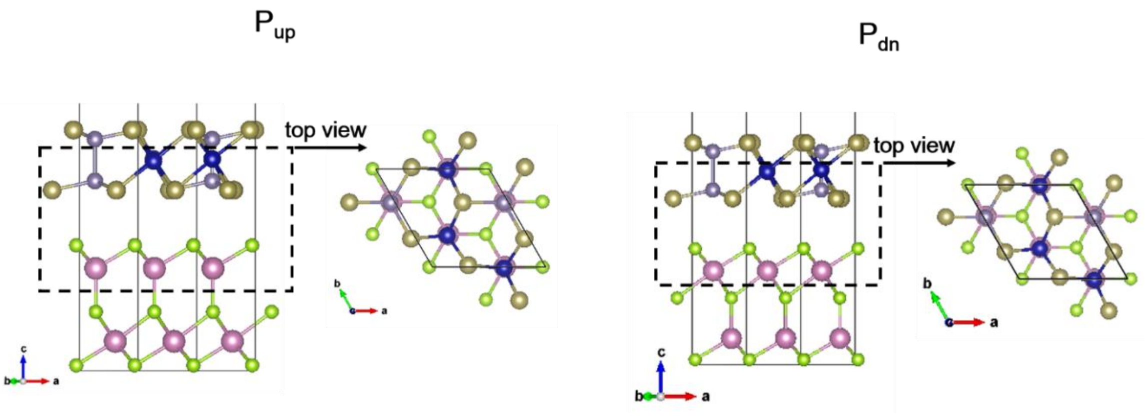

Supplementary Figure 1. The global energy-minimum configurations of In_2Se_3-$Cr_2Ge_2Te_6$ heterostructures for both P_{up}- and P_{dn}-In_2Se_3. The top views show how the interfacial Te atoms of $Cr_2Ge_2Te_6$ contact In_2Se_3: Te atoms are on top of the hollow sites of In_2Se_3, for both P_{up} and P_{dn} cases.

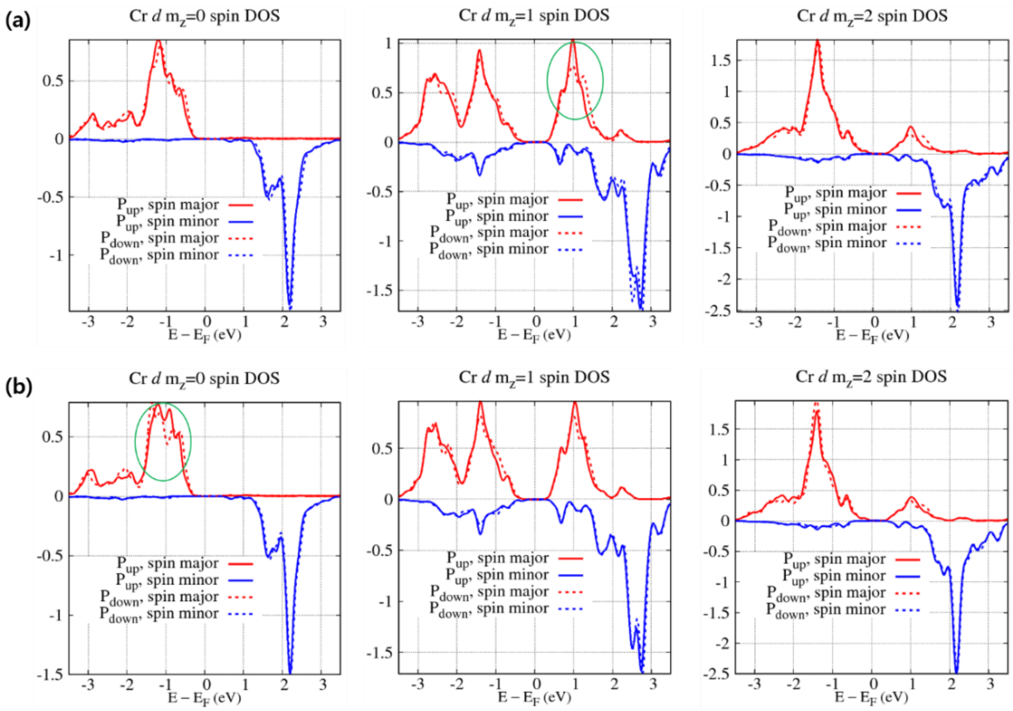

Supplementary Figure 2. Cr spin-resolved *d*-orbital DOS of $Cr_2Ge_2Te_6$/In_2Se_3 with the ferroelectric dipole moment of In_2Se_3 directed upward (solid) and downward (dashed). Upward and downward directions are defined in Fig. 1 in main text. **(a)** and **(b)** correspond to the hollow and top stacking configurations, respectively. The green circle highlights the reduced Cr *d*-orbital DOS by the reversal of In_2Se_3 electric polarization from up to down. As explained in main text, such DOS reduction changes the sign of Cr magnetocrystalline anisotropy energy (MAE) from negative (easy plane) to positive (easy axis), and happens in the *d* m_z=1 conduction band for the hollow configuration, but in the *d* m_z=1 valence band for the top configuration.

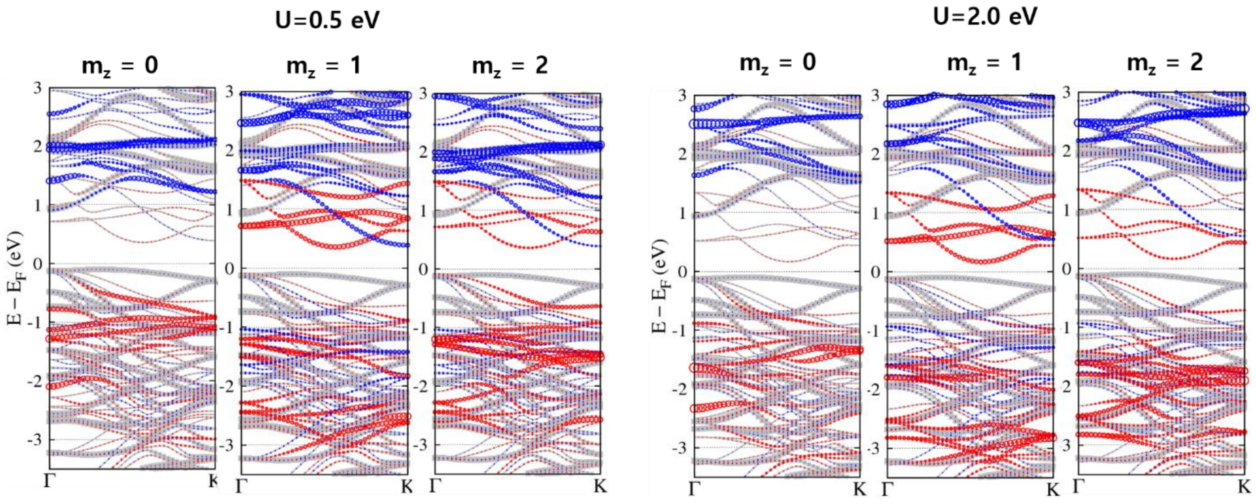

Supplementary Figure 3. U effect of spin-resolved Cr *d*-orbital decomposed band structures for P_{up} heterostructure. With increased U values, the majority spin (red circles) bands shift down, while the minority spin (blue circles) bands shift up, which suppresses the negative value of MAE as discussed in Fig. 2(a) so that the calculated Cr MAE varies from -0.10 meV for U = 0.5 eV to 0.20 meV for U = 2.0 eV.

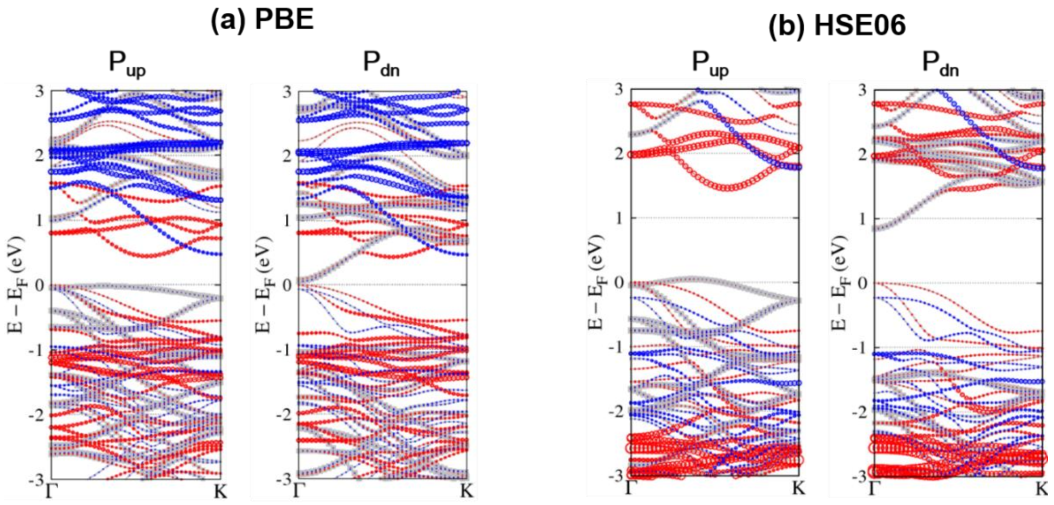

Supplementary Figure 4. The spin-resolved band structures of the In_2Se_3-$Cr_2Ge_2Te_6$ heterostructures for both P_{up}- and P_{dn}-In_2Se_3, calculated by PBE and HSE06. The bands of majority spin are projected by red circles, while the bands of minority spin are projected by blue circles. In the HSE06 calculations (**b**), the key band alignment feature remains the same as PBE calculation results (**a**): while the electric polarization of In_2Se_3 reverses from up to down, the In_2Se_3 conduction band moves down, causing a significant hybridization with the $Cr_2Ge_2Te_6$ conduction band. As seen in (**b**), from P_{up} to P_{dn}, the In_2Se_3 conduction band moves down and hybridizes with $Cr_2Ge_2Te_6$ (as evident by the discontinuous red-dot curve in the range of 1.5~1.8 eV above Fermi level for the P_{dn} case in (**b**)). As discussed in the main text, this feature means from P_{up}- to P_{dn}-In_2Se_3 there is a suppression of negative contribution to MAE, leading to a more positive MAE and out-of-plane anisotropy.

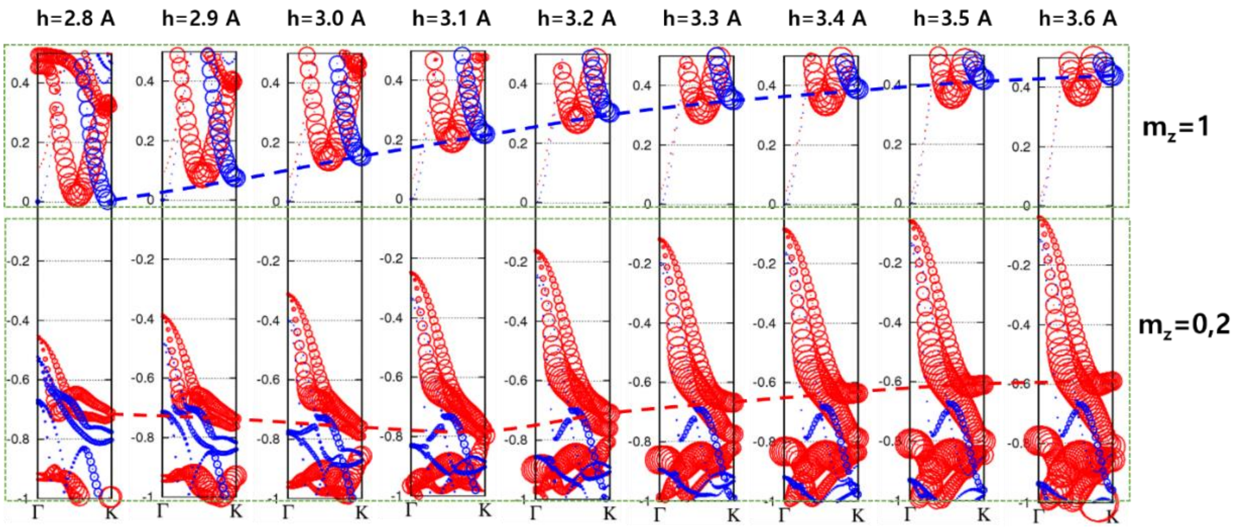

Supplementary Figure 5. Interlayer spacing dependent spin-resolved orbital-decomposed Cr *d*-orbital band structures for top configuration. The circle size of conduction (valence) bands denotes m_z=1 (m_z=0 and 2) contribution. The blue or red dashed line guides a decrease of the direct energy gap with spin-flip ($|\Delta s_z|$=1) near the K point, whose transitions together with $|\Delta m_z|$=1 enhances the positive value of MAE as shown in Fig. 3(b). Note that the top valence bands near K are mainly of $|m_z|$ = 0 and 2, different from conduction band edge.

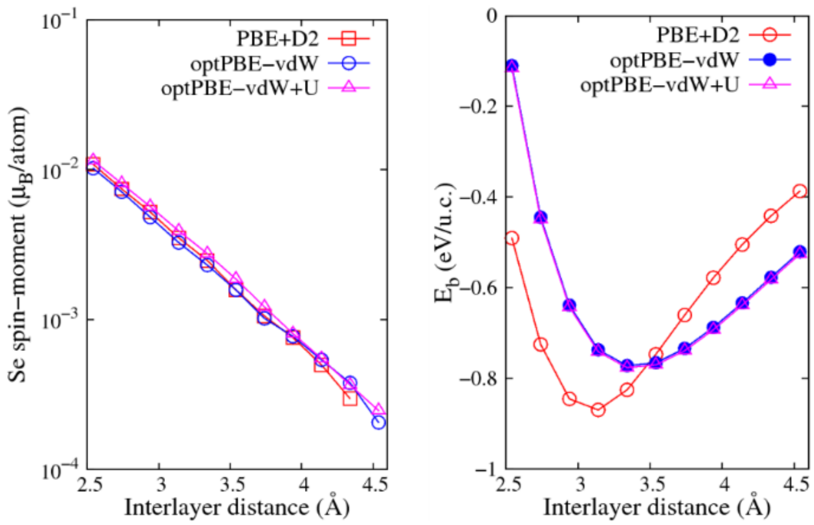

Supplementary Figure 6. Interlayer spacing dependent Se spin moment (left) and interfacial binding energy (right), for different van der Waals density functionals.

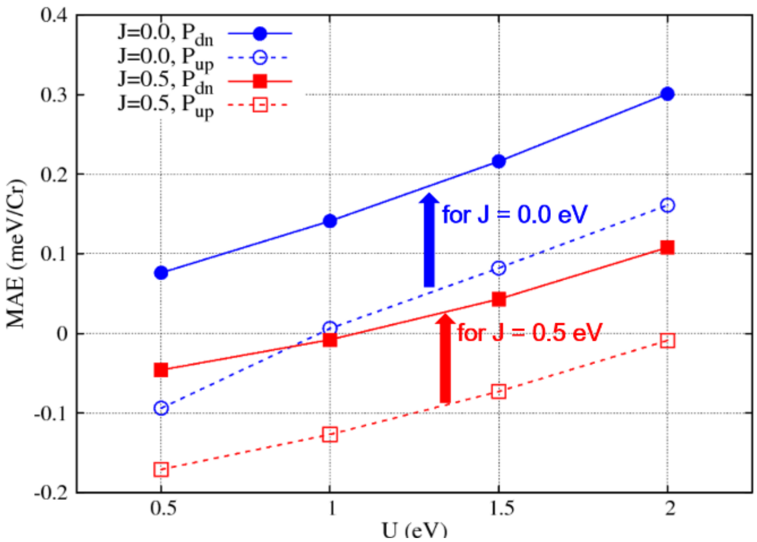

Supplementary Figure 7. The effects of U and J values on the calculated MAE in In_2Se_3-$Cr_2Ge_2Te_6$ heterostructures. The increased U value enhances the out-of-plane anisotropy, while the increased J value enhances the in-plane anisotropy. The out-of-plane anisotropy is enhanced by ~0.15 meV/Cr when the In_2Se_3 dipole is inverted from up to down, for any tested set of U and J. Therefore, even if our adopted values of U and J (U = 0.5 eV, J = 0.0 eV) slightly deviate from the exact description of the real heterostructure samples because of the complex experimental conditions as described in the main text, the reversal of the In_2Se_3 polarization from up to down always strengthens the 2D ferromagnetic order in $Cr_2Ge_2Te_6$. This leads to a general implication: in practice, one can always set a temperature so that 2D ferromagnetism could be found in P_{dn}-In_2Se_3-$Cr_2Ge_2Te_6$ but disappear from P_{up}-In_2Se_3-$Cr_2Ge_2Te_6$, leading to the practical switching experiments at finite temperatures.
